# Evolution trend of soil fertility in tobacco-planting area of Chenzhou, Hunan Province, China

**DOI:** 10.1515/biol-2022-0509

**Published:** 2022-11-28

**Authors:** Yansong Xiao, Yahua Liao, Jianlin Hou, Lijuan Li, Taosha Xu, Fengying Ma, Fahui Yu, Zhipeng Tan, Zhihong He, Hong Jian, Hongguang Li, Bin He

**Affiliations:** Chenzhou Tobacco Production Technology Center of Hunan Tobacco Company, Chenzhou Tobacco Company of Hunan Province, Chenzhou, Hunan 423000, China; Hunan Province Tobacco Company Quality Supervision and Testing Station, Hunan Provincial Tobacco Company, Changsha 410010, China

**Keywords:** tobacco planting, topsoil samples, fertility index, Chenzhou city

## Abstract

In this study, the data of fertility indicators of soil samples (0–20 cm) in 1980s, 2000 and 2015 in Chenzhou city were used, and the soil integrated fertility index (IFI) was calculated. The results showed that the soil pH was decreased, total nitrogen (TN), organic matter (OM), available phosphorus (AP) and potassium (AK), exchangeable calcium (Ca^2+^), magnesium (Mg^2+^) and available copper (Cu) contents were increased, total phosphorus (TP), available sulfur (S) and water-soluble chlorine (Cl^−^) contents were decreased, total potassium (TK), available boron (B), iron (Fe), manganese (Mn) and zinc (Zn) were decreased first and then increased. In 2015, most of the fields were higher in pH, OM, TN, AN, AK, Ca^2+^, Mg^2+^, S, Fe, Mn, Cu and Zn, suitable in B, but lower in TP, AP, TK, available molybdenum (Mo) and Cl^−^. Most of the fields were in the middle grade of IFI in 2000 and 2015, and the mean IFI increased from 0.492 to 0.556 from 2000 to 2015. Thus, for soil improvement, more attention should be paid to adjust soil pH, reduce the application of organic, nitrogen and calcium fertilizers, while increase the fertilizer application of other nutrients.

## 1 Introduction

Soil fertility influences the growth, yield and quality of tobacco [1,2,3,4,5], which is continuously concerned in China. So far, lots of studies have been conducted with many literature studies published on fertility evaluation of tobacco-planting soil; for example, more than 300 literature studies in Chinese could be retrieved by the title or keywords of “tobacco,” “fertility” and “evaluation” in the China National Knowledge Infrastructure (www.dlib.cnki.net/), which almost covered all the tobacco-planting regions in China and at various scales of province, city and county.

Tobacco usually is planted in the drylands with sandy soil texture, but in many areas of southern China, such as Guangdong, Fujian, Jiangxi, Guangxi and south Hunan and Anhui, it is very common that tobacco is planted in rice fields in the high ridge form (no matter what kind of soil texture) and rotated with late rice. Chenzhou city, with a long history of tobacco-planting as early as in 1,593 and where most paddy fields are under tobacco–rice rotation [6], is the most important and typical planting region of flue-cured tobacco with burnt-pure sweet aroma in China [7]. The area of tobacco-planting in Chenzhou is about 2.67 × 10^4^ hm^2^ in recent years, which plays an important role in ensuring the high-quality raw materials supply of the tobacco industry and the local social and economic sustainable development.

Some literature studies were published about tobacco-planting soil nutrient status in Guizhou [8,9,10,11], which played an important guiding role in improving the soil fertility and quality of tobacco-planting fields. However, there are limited fertility indicators (e.g., pH, organic matter (OM), total nitrogen (TN) and available boron) were involved in the above-mentioned studies, and so far, there are few reports reflecting the changes in soil fertility [9]. Two questions are still unclear and should be answered which are concerned with the influences of tobacco-planting on soil fertility: (1) does tobacco-planting really can improve soil fertility? if so, by how much? Also, a new round of tobacco-planting soil improvement is underway in China; it is urgent and helpful to know the status and evolution of soil fertility; therefore, this study was conducted in order to quantitatively analyze the soil fertility of tobacco-planting fields in Chenzhou in order to provide further scientific guidance for fertilization and soil improvement for tobacco-planting in Chenzhou.

## 2 Materials and methods

### 2.1 Data sources of soil fertility indicators

The data of soil fertility indicators used in this study came from three periods, the 2nd national soil survey conducted in the 1980s [12], and tobacco-planting soil surveys conducted in 2000 and 2015, which included 350, 746 and 1,055 soil samples, respectively. The obtained data of the 1980s were the statistic information of all soil samples, and no data of each sample is available.

According to the historical records, the soil sample of the plough layer in each field was collected randomly at 5–8 points with stainless steel soil drill and then mixed completely. The measured soil properties (soil fertility indicators) included OM, TN, total phosphorous (TP), total potassium (TK), available nitrogen (AN), available phosphorous (AP), available potassium (AK), available boron (B), available iron (Fe), available manganese (Mn), available copper (Cu), available zinc (Zn) and available molybdenum (Mo) for all soil samples in the three periods, and pH (H_2_O), exchangeable calcium (Ca^2+^), exchangeable magnesium (Mg^2+^), available sulfur (S), and water-soluble chlorine (Cl^−^) for the soil samples in 2000 and 2015. The detailed determination methods for soil fertility indicators could be found in related literature studies [13,14].

### 2.2 Quantitative assessment of soil fertility

There are various methods for the assessment of soil fertility; in this study, soil integrated fertility index (IFI) was used to evaluate soil fertility, and IFI was calculated with the fuzzy mathematics method [15]; first, the membership function types and inflection points of indicators were determined; second, the membership values of the indicators were calculated; third, the weights of the indicators were determined, and finally, IFI was calculated for soil samples according to the following formula:

(1) \text{IFI}=\sum ({W}_{i}\times {N}_{i}),]

where *W*_*i*_ stands for the weight of indicator *i* and *N*_*i*_ for the membership value of indicator *i*. IFI is ranged from 0 to 1. The higher the IFI value, the higher the soil fertility. Generally, IFI is divided into five grades according to the equidistant method [16,17]: ≥0.80 (higher), 0.6–0.8 (high), 0.4–0.6 (Middle), 0.2–0.4 (low) and <0.2 (lower).

#### 2.2.1 Grading standards of fertility indicators

There are many reports available in China on the grading standards of soil fertility indicators for tobacco-planting fields. In this study, the indicators were divided into 4 or 5 grades as in Table 1 based on the actual situation of tobacco-planting soils in Hunan Province [18,19] and the corresponding grading of tobacco-planting soils in neighboring areas of Hunan Province [20,21,22,23,24,25,26].

**Table 1:** Grading standards of soil fertility indicators for tobacco-planting fields

Fertility indicator	Grade				
	Very low	Low	Suitable	High	Very high
pH	<5.0	5.0–5.5	5.5–7.0	7.0–7.5	≥7.5
SOM	<10	10–15	15–30	30–40	≥40
TN	<0.5	0.5–1	1–2	2–2.5	≥2.5
AN	<65	65–100	100–180	180–240	≥240
TP	<0.5	0.5–1	1–1.5	≥1.5	
AP	<10	10–15	15–30	30–40	≥40
TK	<10	10–15	15–20	20–25	≥25
AK	<80	80–150	150–220	220–350	≥350
Ca^2+^	<3	3–6	6–10	10–18	≥18
Mg^2+^	<0.5	0.5–1.0	1.0–1.6	1.6–3.2	≥3.2
S	<10	10–16	16–30	30–50	≥50
B	<0.15	0.15–0.3	0.3–0.6	0.6–1.0	≥1.0
Fe	<2.5	2.5–4.5	4.5–10	10–60	≥60
Mn	<5	5–10	10–20	20–40	≥40
Cu	<0.2	0.2–0.5	0.5–1.0	1.0–3.0	≥3.0
Zn	<0.5	0.5–1.0	1.0–2.0	2.0–4.0	≥4.0
Mo	<0.1	0.1–0.15	0.15–0.2	0.2–0.3	≥0.3
Cl^−^	<5	5–10	10–30	30–40	≥40

Notes: in the first column, SOM, TN, TP, TK, g/kg; AN, AP, AK, S, B, Fe, Mn, Cu, Zn, Mo, Cl^−^, mg/kg; Ca^2+^, cmol(1/2Ca^2+^)/kg; Mg^2+^, cmol(1/2Mg^2+^)/kg. The same is below.

#### 2.2.2 Calculation of membership value of fertility indicator

The membership function types of the fertility indicators were determined according to their effects on the growth, yield and quality of tobacco, among which, pH, OM, TN, AN and Cl^−^ were belonged to the parabolas type, while TP, AP, TK, AK, Ca, Mg, S, B, Fe, Mn, Cu, Zn and Mo belonged to the S type [15,27]. Combining with the actual situation of tobacco growing soils in Chenzhou [8,9,10,11,17], the turning points of membership function of each fertility indicator were determined according to expert experience and related literature studies published [5,16,17,18,19,22,26,28] (Table 2; *x*_1_, lower limiting value; *x*_2_, upper limiting value; *x*_3_, lower optimal value; and *x*_*4*_, upper optimal value).

**Table 2:** Membership function types and turning points of soil fertility indicators

Fertility indicator	Membership function type	Lower limit, *x*_1_	Lower optimal, *x*_3_	Upper optimal, *x*_4_	Upper limit, *x*_2_
pH	Parabolas	5	5.5	7	8
SOM	Parabolas	15	20	35	45
TN	Parabolas	0.5	1	2	2.5
AN	Parabolas	65	100	180	240
Cl^−^	Parabolas	5	10	30	40
TP	S	0.5			1.5
AP	S	10			40
TK	S	10			25
AK	S	80			350
Ca	S	3			20
Mg	S	0.5			4
S	S	16			30
B	S	0.2			1.5
Fe	S	4.5			70
Mn	S	10			50
Cu	S	0.5			4
Zn	S	1			5
Mo	S	0.15			0.4

The membership functions of S-type and parabolic indicators were calculated as follows:

\text{S}\hspace{.25em}\text{type}\hspace{.25em}f(xi)=\left\{\begin{array}{ll}0.1, {x}_{i}\lt {x}_{1},\\ 0.1+0.9\times ({x}_{i}-{x}_{1})/({x}_{2}-{x}_{1}), {x}_{1}\le {x}_{i}\lt {x}_{2},\\ 1, {x}_{i}\ge {x}_{2},\end{array}\right.]

\text{Parabolas}\hspace{.25em}\text{type}\hspace{.25em}f(xi)=\left\{\begin{array}{ll}0.1, {x}_{i}\lt {x}_{1},{x}_{i}\ge {x}_{2},\\ 0.1+0.9\times ({x}_{i}-{x}_{1})/({x}_{3}-{x}_{1}), {x}_{1}\le {x}_{i}\lt {x}_{3},\\ 1, {x}_{3}\le {x}_{i}\lt {x}_{4},\\ 1.0-0.9\times ({x}_{i}-{x}_{4})/({x}_{2}-{x}_{4}) {x}_{4}\le {x}_{i}\lt {x}_{2}.\end{array}\right.]

#### 2.2.3 Determination of weights of indicators

The weights of indicators were determined by principal component analysis (PCA), which is commonly used in soil fertility and quality evaluation [21,29,30]. In order to avoid the appearance of a negative value in weight, the measured values of pH, OM, TN, TP, TK, AN, AP, AK, AS, Ca, Mg, S, B, Fe, Cu, Zn, Mo and Cl^−1^ were standardized using *Z*-score standardization method, while those of Mn were standardized using the negative range normalization method in SPSS software [31,32]. The KMO test coefficient obtained was 0.727, indicating that the data structure was good and the linear correlation between the data was satisfied, which could be used for principal component analysis, while the *p* value of Bartlett’s test was less than 0.001, rejecting the null hypothesis, indicating that the data could be extracted by principal components. The obtained weights of indicators are shown in Table 3, and the detailed routine for the acquisition of indicator weights was not listed here.

**Table 3:** Weight values of soil fertility indicators for tobacco-planting fields in Chenzhou

Indicator	pH	OM	TN	TP	TK	AN	AP	AK	Ca
Weight	0.031	0.075	0.065	0.061	0.026	0.058	0.066	0.065	0.032
Indicator	Mg	S	B	Fe	Mn	Cu	Zn	Mo	Cl
Weight	0.039	0.072	0.067	0.014	0.017	0.115	0.114	0.044	0.039

### 2.3 Data processing and statistics

Microsoft Excel 2016 and IBM Statistics SPSS 22.0 software were used for the statistical analysis of the data, and Duncan test method (*p* < 0.05) was used for the analysis of variance and multiple comparisons [31,32].

## 3 Results

### 3.1 Statistics and comparison of soil fertility indicators

Table 4 shows the statistical results of the indicators in the three periods. From the average values of the indicators, it is shown in Table 3 that pH was reduced insignificantly from 7.18 in 2000 to 6.99 in 2015 (Sig. = 0.198, two-tailed, the same below). OM showed an increasing tendency between 1980s and 2015, which increased by 18.22% from 1980 to 2000 and significantly by 4.64% from 2000 to 2015 (Sig. = 0.008). TN also showed an increasing tendency between 1980 and 2015, which increased by 44.51% from 1980 to 2000 and insignificantly by 1.14% from 2000 to 2015 (Sig. = 0.680). TP showed a decreasing tendency from 1980 to 2015, decreased by 31.39% from the 1980s to 2000 and significantly by 2.13% from 2000 to 2015 (Sig. = 0.001). TK decreased first then increased within the 1980s–2015, decreased by 53.05% from the 1980s to 2000 and then increased significantly by 16.45% from 2000 to 2015 (Sig. = 0.000). AN increased first then decreased within the 1980s–2015, increased by 51.90% from 1980s to 2000 and then decreased significantly by 9.69% from 2000 to 2015 (Sig. = 0.000). AP and AK both showed an increasing tendency within 1980–and 2015, which increased by 216.85 and 57.64% from the 1980s to 2000 and significantly by 29.36 and 70.36% from 2000 to 2015 (Sig. = 0.000). Ca, Mg and Cu all showed an increasing tendency from 2000 to 2015, which increased significantly by 157.31%, 20.44 and 16.58% (*Sigs*. of Ca and Mg = 0.000, Sig. Cu = 0.003). S and Cl^−^ both showed a decreasing tendency from 2000 to 2015, which decreased significantly by 24.08 and 65.00% (both *Sigs*. = 0.000). B, Fe, Mn and Zn all decreased first then increased within the 1980s–2015, decreased by 57.50, 17.87, 33.88 and 0.28% from 1980s to 2000 and then increased significantly by 223.53, 85.82, 34.52 and 23.68%, respectively, from 2000 to 2015 (*Sigs*. of B, Fe and Mn = 0.000, Sig. of Zn = 0.008). Mo showed a decreasing tendency within 1980–2015 and decreased by 16.00% and significantly by 23.81% (Sig. = 0.015).

**Table 4:** Statistic information of soil fertility indicators in 2000 and 2015 in Chenzhou

Indicators	1980s (*n* = 350)			2000 (*n* = 746)					2015 (*n* = 1,055)				
	Mean ± S.D.	C.V. (%)	Grade	Mean ± S.D.	Grade	C.V. (%)	Skewness	Kurtosis	Mean ± S.D.	Grade	C.V. (%)	Skewness	Kurtosis
pH	/	/	/	7.18 ± 0.94a	High	13.09	−0.98	−0.26	6.99 ± 0.93a	Suitable	13.30	−0.97	−0.34
OM	38.8 ± 2.70	6.96	High	45.87 ± 13.98A	Very high	30.48	0.03	0.03	48.00 ± 14.37B	Very high	29.94	0.46	0.51
TN	1.82 ± 0.15	8.24	Suitable	2.63 ± 0.74a	Very high	28.14	0.09	−0.03	2.66 ± 0.71a	Very high	26.69	0.89	1.40
TP	1.37 ± 0.17	12.41	Suitable	0.94 ± 0.29A	Low	30.85	0.33	1.05	0.92 ± 0.28B	Low	30.43	0.68	1.25
TK	23.30 ± 1.61	6.91	High	10.94 ± 2.99A	Low	27.33	0.47	0.28	12.74 ± 3.83B	Low	30.06	0.41	0.10
AN	147.97 ± 7.87	5.32	Suitable	224.76 ± 68.10A	High	30.30	0.37	0.74	202.98 ± 54.04B	High	26.62	0.32	1.78
AP	8.90 ± 1.00	11.24	Very low	28.20 ± 12.81A	Suitable	45.43	1.73	11.17	36.48 ± 17.74B	High	48.63	2.33	10.16
AK	76.60 ± 5.63	7.35	Very low	120.75 ± 60.02A	Low	49.71	4.79	46.4	205.71 ± 87.47B	Suitable	42.52	0.56	1.14
Ca	/	/	/	12.93 ± 5.77A	high	44.62	0.86	1.27	33.27 ± 23.27B	very high	69.94	8.26	107.76
Mg	/	/	/	1.37 ± 0.78A	Suitable	56.93	0.88	0.89	1.65 ± 1.04B	High	63.03	0.62	−1.00
S	/	/	/	51.92 ± 24.48a	Very high	47.15	1.57	6.91	39.42 ± 35.81b	High	90.84	1.25	2.02
B	0.40 ±	/	Suitable	0.17 ± 0.10A	Low	58.82	1.58	6.75	0.55 ± 0.18B	Suitable	32.73	1.23	1.23
Fe	93.61 ±	/	Very high	76.88 ± 49.20A	Very high	64.00	1.30	1.38	142.86 ± 89.55B	Very high	62.68	2.94	12.37
Mn	37.37 ±	/	High	24.71 ± 29.99A	high	121.37	5.51	46.82	33.24 ± 31.52B	High	94.83	12.66	190.18
Cu	3.74 ±	/	Very high	4.36 ± 4.28A	very high	119.22	13.81	240.83	4.70 ± 5.31B	Very high	119.59	15.65	318.23
Zn	3.60 ±	/	High	3.59 ± 5.37A	high	123.17	8.12	85.72	4.44 ± 9.77B	Very high	207.87	1.05	1.88
Mo	0.25±	/	High	0.21 ± 0.28a	High	133.33	9.95	156.99	0.16 ± 0.23b	Low	143.75	8.18	124.05
Cl^−^	/	/	/	18.49 ± 10.34A	Suitable	57.44	1.12	3.89	6.30 ± 9.84B	Low	156.19	2.72	11.96

Note: (1) Data defaulted; (2) total sample numbers of Ca^2+^ and Mg^2+^ in 2000 is 314; (3) values in the same line followed by different uppercase or lowercase letters are significantly different at the 0.01 or 0.05 level: the same as below.

In the 1980s, OM, TN, TK, AN and AK belonged to the low variation (C.V. < 10%,), while TP and AP belonged to the moderate variation (C.V. = 10–100%). In 2000, pH, OM, TN, TP, TK, AN, AP, AK, Ca, Mg, S, B, Fe and Cl^−^ belonged to moderate middle variation, while Mn, Cu, Zn and Mo belonged to the strong variation (C.V. > 100%). In 2015, pH, OM, TN, TP, TK, AN, AP, AK, Ca, Mg, S, B, Fe and Mn remained in the moderate middle variation, Cu, Zn and Mo remained in the strong variation (C.V. > 100%), while Cl^−^ changed from the moderate variation to the strong one. It also can be seen from Table 4 that, in the values of C.V.s (%), pH, OM, TN, TP, TK, AN, AP, AK, Mg, Fe, Cu and Mo changed smaller (all lower than 15%), while Ca, S, Zn and Cl increased greatly (56.74, 92.67, 68.78 and 171.90%, respectively), and B and Mn decreased greatly (44.36 and 21.87%, respectively).

Table 5 shows the statistical information on fertility indicators in each grade in 2000 and 2015. It can be seen from tobacco-planting suitability, in 2000 and 2015, 26.81 and 26.73% of the samples were suitable in pH, 65.42 and 62.37% of the samples were higher in pH (including high and very higher grades, the same as below), and 7.77 and 10.90% of the samples were lower in pH (including low and very low grades, the same below). Most soil samples are high in OM, TN, AN, AK, Ca^2+^, S, B, Fe, Cu and Zn, among which, 86.06 and 90.33% of the samples were higher in OM, 79.36 and 83.13% of the samples were higher in TN, 75.60 and 64.55% of the samples were higher in AN, 82.44 and 27.30% of the samples were lower in AK, 67.52 and 85.12% of the samples were higher in Ca^2+^, 84.05 and 55.55% of the samples were higher in S, 84.05 and 55.55% of the samples were higher in B, 99.20 and 100% of the samples were higher in Fe, 99.06 and 99.72% of the samples were higher in Cu, and 70.64 and 77.73% of the samples were higher in Zn. Table 5 also shows that obvious proportions of soil samples were lower in TP, AP, Mg^2+^, Mo and Cl^−^ in 2000 and 2015, among which, 90.75 and 79.81% of the samples were lower in TK, 60.05 and 59.24% of the samples were lower in TP, 38.61 and 60.00% of the samples were higher in AP, 40.13 and 32.04% of the samples were lower in Mg^2+^, 49.20 and 66.54% of the samples were lower in Mo, and 21.18 and 76.30% of the samples were lower in Cl^−^.

**Table 5:** Grade statistic information of soil fertility indicators in 2000 and 2015 in Chenzhou

Indicators	Year	Very low		Low		Suitable		High		Very high		Total	
		No.	%	No.	%	No.	%	No.	%	No.	%	No.	%
pH	2000	18	2.41	40	5.36	200	26.81	61	8.18	427	57.24	746	100
	2015	45	4.27	70	6.64	282	26.73	134	12.70	524	49.67	1,055	100
SOM	2000	1	0.13	5	0.67	98	13.14	135	18.10	507	67.96	746	100
	2015	1	0.09	0	0.00	101	9.57	224	21.23	729	69.10	1,055	100
TN	2000	0	0.00	7	0.94	147	19.71	144	19.30	448	60.05	746	100
	2015	0	0.00	0	0.00	178	16.87	278	26.35	599	56.78	1,055	100
AN	2000	3	0.40	16	2.14	163	21.85	278	37.27	286	38.34	746	100
	2015	1	0.09	17	1.61	356	33.74	438	41.52	243	23.03	1,055	100
TP	2000	59	7.91	389	52.14	271	36.33	27	3.62	/	0.00	746	100
	2015	73	6.92	552	52.32	415	39.34	15	1.42	/	0.00	1,055	100
AP	2000	35	4.69	57	7.64	366	49.06	169	22.65	119	15.95	746	100
	2015	30	2.84	73	6.92	319	30.24	231	21.90	402	38.10	1,055	100
TK	2000	290	38.87	387	51.88	63	8.45	6	0.80	0	0.00	746	100
	2015	206	19.53	636	60.28	167	15.83	34	3.22	12	1.14	1,055	100
AK	2000	131	17.56	484	64.88	100	13.40	22	2.95	9	1.21	746	100
	2015	54	5.12	234	22.18	337	31.94	372	35.26	58	5.50	1,055	100
Ca^2+^	2000	2	0.64	29	9.24	71	22.61	160	50.96	52	16.56	314	100
	2015	2	0.19	47	4.45	108	10.24	234	22.18	664	62.94	1,055	100
Mg^2+^	2000	30	9.55	96	30.57	72	22.93	110	35.03	6	1.91	314	100
	2015	72	6.82	266	25.21	248	23.51	375	35.55	94	8.91	1,055	100
S	2000	5	0.67	23	3.08	91	12.20	257	34.45	370	49.60	746	100
	2015	10	0.95	81	7.68	378	35.83	365	34.60	221	20.95	1,055	100
B	2000	325	43.57	350	46.92	68	9.12	3	0.40	0	0.00	746	100
	2015	0	0.00	39	3.70	656	62.18	329	31.18	31	2.94	1,055	100
Fe	2000	0	0.00	1	0.13	5	0.67	368	49.33	372	49.87	746	100
	2015	0	0.00	0	0.00	0	0.00	145	13.74	910	86.26	1,055	100
Mn	2000	36	4.83	148	19.84	282	37.80	170	22.79	110	14.75	746	100
	2015	49	4.64	88	8.34	278	26.35	362	34.31	278	26.35	1,055	100
Cu	2000	0	0.00	1	0.13	6	0.80	156	20.91	583	78.15	746	100
	2015	0	0.00	1	0.09	2	0.19	223	21.14	829	78.58	1,055	100
Zn	2000	0	0.00	24	3.22	195	26.14	389	52.14	138	18.50	746	100
	2015	2	0.19	28	2.65	205	19.43	511	48.44	309	29.29	1,055	100
Mo	2000	223	29.89	144	19.30	114	15.28	126	16.89	139	18.63	746	100
	2015	507	48.06	195	18.48	118	11.18	102	9.67	133	12.61	1,055	100
Cl^−^	2000	64	8.58	94	12.60	512	68.63	50	6.70	26	3.49	746	100
	2015	683	64.74	122	11.56	218	20.66	13	1.23	19	1.80	1,055	100

### 3.2 Statistics and comparison of soil IFIs

Tables 6 and 7 present the general and grade statistical results of soil IFIs of tobacco-planting fields, respectively. Tables 6 and 7 show that there was no sample with IFI < 0.2 or ≥0.8 both in 2000 and in 2015, IFI were within 0.230–0.740 with a mean of 0.492 in 2000 and 0.320–0.760 with a mean of 0.556 in 2015, and both covered the lower (<0.2), middle (0.2–0.4) and higher grades (0.4–0.6). However, it also can be seen that the average IFI significantly increased by 13.01% (Sig. = 0.000) from 2000 to 2015. IFI in both 2000 and 2015 belonged to the moderate variation, negative skew distribution in 2000 but near normal distribution in 2015, and flat peak distribution in 2000 and 2015 [31,32]. It is shown in Table 7 that the samples with IFI within 0.4–0.6 were most both in 2000 (77.35%) and 2015 (70.52%), and the sample proportions with IFI within 0.2–0.4 and 0.4–0.6 were decreased from 12.87% in 2000 to 1.61% in 2015 and from 77.35% in 2000 to 70.52% in 2015, respectively, while the sample proportion with IFI within 0.6–0.8 increased from 9.79% in 2000 to 27.87% in 2015.

**Table 6:** Statistic information of soil IFI*s* in 2000 and 2015 in Chenzhou

Region	Year	Sample No.	Min	Max	Mean ± S.D.	C.V. (%)	Skewness	Kurtosis
Guiyang	2000	447	0.254	0.715	0.486 ± 0.077A	15.821	0.230	−0.227
	2015	560	0.319	0.756	0.559 ± 0.076B	13.65	−0.014	−0.087
Jiahe	2000	100	0.237	0.736	0.492 ± 0.086A	17.512	0.123	0.353
	2015	110	0.408	0.737	0.554 ± 0.073B	13.10	0.174	−0.504
Yongxing	2000	73	0.229	0.735	0.498 ± 0.094A	18.901	−0.121	0.368
	2015	115	0.375	0.691	0.547 ± 0.063B	11.57	−0.031	−0.345
Anren	2000	35	0.369	0.702	0.520 ± 0.084A	16.149	0.320	−0.338
	2015	100	0.438	0.711	0.563 ± 0.061B	10.76	0.164	−0.334
Yizhang	2000	29	0.371	0.613	0.486 ± 0.069A	14.082	0.236	−0.974
	2015	96	0.351	0.725	0.548 ± 0.081B	14.70	−0.007	−0.379
Suxian	2000	20	0.396	0.623	0.508 ± 0.071a	13.959	−0.018	−0.979
	2015	45	0.347	0.666	0.546 ± 0.077b	14.10	−0.668	0.141
Beihu	2000	12	0.419	0.608	0.526 ± 0.064a	12.220	−0.533	−1.010
	2015	17	0.419	0.674	0.587 ± 0.066a	11.30	−0.740	1.100
Linwu	2000	30	0.350	0.637	0.511 ± 0.087a	17.076	−0.186	−0.957
	2015	12	0.349	0.654	0.548 ± 0.085a	15.55	−0.942	1.680
Total	2000	746	0.230	0.740	0.492 ± 0.080A	16.26	0.150	−0.087
	2015	1,055	0.320	0.760	0.556 ± 0.074B	13.25	−0.040	−0.101

Lowercase and majuscule indicate significant differences at the 0.05 and 0.01 levels, respectively.

**Table 7:** Grade statistic information of soil IFI*s* in 2000 and 2015 in Chenzhou

Region	Year	IFI									
		Highest (≥0.8)		Higher (0.6–0.8)		Middle (0.4–0.6)		Lower (0.2–0.4)		Lowest ( < 0.2)	
		No.	%	No.	%	No.	%	No.	%	No.	%
Guiyang	2000	0	0	36	8.05	348	77.85	63	12.00	0	0
	2015	0	0	158	28.21	393	70.18	9	1.61	0	0
Jiahe	2000	0	0	9	6.00	79	79.00	12	12.00	0	0
	2015	0	0	25	22.73	84	76.36	1	0.91	0	0
Yongxing	2000	0	0	10	13.70	54	73.97	9	12.33	0	0
	2015	0	0	33	28.00	82	71.30	0	0	0	0
Anren	2000	0	0	5	14.29	27	77.14	3	8.57	0	0
	2015	0	0	28	28.00	72	72.00	0	0	0	0
Yizhang	2000	0	0	2	6.90	24	82.76	3	10.34	0	0
	2015	0	0	29	30.21	64	66.67	3	3.13	0	0
Suxian	2000	0	0	2	10.00	17	85.00	1	5.00	0	0
	2015	0	0	11	24.44	31	68.89	3	6.67	0	0
Beihu	2000	0	0	2	16.67	10	83.33	0	0	0	0
	2015	0	0	7	41.18	10	58.82	0	0	0	0
Linwu	2000	0	0	7	23.33	18	60.00	5	16.67	0	0
	2015	0	0	3	25.00	8	66.67	1	8.33	0	0
Total	2000	0	0	73	9.79	577	77.35	96	12.87	0	0
	2015	0	0	294	27.87	744	70.52	17	1.61	0	0

From 2000 to 2015, IFI was significantly increased by 13.00% in total (Sig. 000), 14.94% in Guiyang (Sig. = 0.000), 12.70% in Jiahe (Sig. = 0.000), 9.74% in Yongxing (*Sigs*. = 0.000), 8.24% in Anren (Sig. = 0.001), 12.72% in Yizhang (Sig. = 0.001) and 7.47% in Suxian (Sig. = 0.024), while IFI was increased insignificantly by 11.56 and 7.13% in Beihu (Sig. = 0.147) and in Linwu (Sig. = 0.248). Difference significance test results also showed that in 2000 significant difference in IFI was only founded between Guiyang with Linwu (Sig. = 0.009) and Anren (Sig. = 0.014), and no significant difference among the other regions (Sig. = 0.052–0.977 with a mean of 0.330), while in 2015 significant difference in IFI was only founded between Anren and Suxian (Sig. = 0.047), and no significant difference among the other regions (Sig. = 0.060–0.978 with a mean of 0.442).

Table 7 shows that in 2000 and 2015, the numbers of samples were the most in the middle grade of IFI among the total samples, which were 77.35 and 70.52% in total, 77.85 and 70.18% in Guiyang, 79.00 and 76.36% in Jiahe, 73.97 and 71.30% in Yongxing, 77.14 and 72.00% in Anren, 82.76 and 66.67% in Yizhang, 85.00 and 68.89% in Suxian, 83.33 and 58.82% in Beihu, and 60.00 and 66.67% in Linwu. Table 7 also shows that the proportion of samples in the higher grade of IFI was increased from 9.79% in 2000 to 27.87% in 2015 in total, while that of samples in the lower grade of IFI were decreased from 12.87% in 2000 to 1.61% in 2015 in total.

## 4 Discussion

Soil fertility assessment is one of the most basic works in soil science research, but it is very important for crop planting; therefore, the latest literature studies could be found even now [33,34,35,36,37]. Meanwhile, soil fertility evaluation of tobacco planting also has been reported more so far; however, the fertility indicators mainly involve pH, OM, TN, AN, TP, AP, TK, AK, Ca^2+^, Mg^2+^, trace elements of B, Fe, Mg, Cu and Zn, and Cl^−^; in our study, besides the above indicators, S and Mo were also added; thus, it should be said that the fertility indicators are more complete in our study, which would enable the obtained results are closer to the reality and more feasible in guiding the scientific fertilization and soil improvement. It should be pointed out that CEC is also one of the most important indicators of soil fertility; however, as in many studies of soil fertility assessment conducted in China, the determined indicators of soil fertility usually include soil pH and the contents of OM and main nutrients [1,2,3,4,5], which can not only understand the real state of in each index (whether suitable or not for high-quality tobacco planting) in order to guide the reasonable fertilization and soil improvement, but also can evaluate soil comprehensive fertility based on these indicators. The reason why CEC is rarely used is that CEC is a comprehensive indicator of soil fertility, it can only indicate the general level of soil fertility, but cannot provide the real information on soil pH and the contents of OM and nutrient contents, thus cannot guide the reasonable fertilizer application and soil improvement; therefore, CEC was also not used in our study. Meanwhile, soil pH and OM content are the factors that can influence CEC, so CEC can be omitted when pH and OM are used for soil fertility assessment. We add some content in the second revised manuscript. Meanwhile, the Ca/K and Ca/Mg ratios should be considered in the assessment of soil fertility for tobacco-planting, which is important to instruct the scientific application of potassium by reflecting the nutrient antagonism in soils, but currently, there are no threshold values or grade classifications of Ca/K and Ca/Mg for tobacco, so these two ratios were also not used in this study.

As shown in Table 5, 65.42 and 62.37% of the samples were higher in pH (≥7.0) in 2000 and 2015, respectively. The high value of soil pH of tobacco-planting fields in Chenzhou could be attributed to the application of superphosphate fertilizer and the habit of local farmers using fired soil to improve soil quality [38,39], and it may also be related to that tobacco-planting fields in Chenzhou are mostly located in the limestone hill and mountainous area [10], which also resulted in the increases of Ca^2+^ and Mg^2+^ from 2000 to 2015, increased significantly by 157.31 and 20.44%, respectively (Table 4).

OM was increased from 38.8 g/kg in the 1980s to 45.87 g/kg in 2000 and to 48.00 g/kg in 2015, the increase was decided by tobacco-rice rotation, straw returning to the field and organic fertilizer application [11,40,41]. AN increased from 147.97 mg/kg in the 1980s to 224.76 mg/kg in 2000 and then decreased to 202.98 mg/kg in 2015, the decrease in AN from 2000 to 2015 is because the higher content of AN in 2000 is unsuitable (suitable grade is 100–180 mg/kg) for the high-quality tobacco [25], and thus, the applied amount of nitrogen fertilizer was reduced gradually [42]. AP increased from 8.90 mg/kg in the 1980s to 28.20 mg/kg in 2000 and to 36.48 mg/kg in 2015; the remarkable continuous increase could be attributed to the long-term excessive application of phosphatic fertilizer by farmers in China due to the cheap price and yield-increase effect [43,44]. AK increased from 76.6 mg/kg in the 1980s to 120.75 mg/kg in 2000 and to 205.71 mg/kg in 2015, the continuous significant increase was contributed by the large amount application of potassium to guarantee the high-quality tobacco leaves usually with high K content [1,2,3,4,5]. S deceased from 51.92 mg/kg in 2000 to 39.42 mg/kg in 2015, which could be attributed to the reduced use of potassium sulfate for tobacco-planting because it caused soil acidification, and usually high content of S would worsen the quality of tobacco leaves [45,46]. Cl^−^ decreased from 18.49 mg/kg in 2000 to 6.30 mg/kg in 2015, which could be attributed to the worry that high Cl^−^ content could severely deteriorate the quality of tobacco leaves, so chlorine fertilizer is seldom used for tobacco-planting in many regions [47]. Mo decreased from 0.25 mg/kg in the 1980s to 0.21 mg/kg in 2000 and to 0.16 mg/kg in 2015, which may be related to little concern about Mo and little literature on Mo fertilizer application for tobacco-planting in Hunan [48]. Cu increased from 3.74 mg/kg in the 1980s to 4.36 mg/kg in 2000 and to 4.70 mg/kg in 2015, the increase, on the one hand, may be related to the application of livestock and poultry manure which usually containing Cu [49,50], and on the other hand, may be related to the higher Cu content in paddy soil itself [51,52], as for the changes of B, Zn, Mn and Fe, which decreased from the 1980s to 2010 and then increased from 2000 to 2015, which could be attributed to the gradual application of related trace fertilizers in the farmlands [53].

Li et al. [23] compared soil fertility indicators tobacco-growing areas in Kunming in southwest China in 2010 and 2020 and found that from 2010 to 2020, soil pH decreased by 0.15 units. Soil SOM, N, P and K increased by 1.87 g/kg, 7.21 mg/kg, 5.17 mg/kg and 53.05 mg/kg, respectively; the overall soil fertility increased, and these findings are generally similar to the results in our study.

The change of climate may influence the changes in soil chemical properties, so we analyzed the correlation between the mean annual temperature (T) and precipitation (P) with years from 2000 and 2015, and the results showed that the ranges of the two parameters were 17.9–19.1°C and 1069–1854 mm with the means of 18.6°C and 1450 mm, respectively; but there was no significant correlation between T and P with year. The Pearson coefficients were 0.499 (*p* = 0.082) and 0.224 (*p* = 0.462), respectively, which means it is hard to clarify the influence of climate change on the change of soil fertility instructors.

Soil fertility affects or determines the growth, yield and quality of tobacco; it also determines the economic benefits of local tobacco-planting farmers, so more and great concerns have been paid continuously to soil improvement in the tobacco-planting regions with higher input and sufficient guarantee, almost all the tobacco-planting regions in China have formulated and implemented the technical regulation of flue-cured tobacco planting, which enable the same measures adopted for tobacco-planting in ploughing, ridging, fertilization, irrigation, film mulching, etc. It may homogenize the soil fertility of tobacco-planting fields in a large region, so no significant difference in IFI was found in this study between most tobacco-planting regions in Chenzhou, in which significant difference in IFI was only founded between Guiyang with Linwu (Sig. = 0.009) and Anren (Sig. = 0.014) in 2000 and between Anren and Suxian (Sig. = 0.047) in 2015. Our study also showed the increasing tendency of soil IFI of tobacco-planting fields in Chenzhou, it was not only proved by the increases in the contents of OM, AN, AP and AK from 1980 to 2000 (Table 1), meanwhile, but also proved by the proportion of fields with the lower grade of IFI was decreased from 12.87% in 2000 to 1.61% in 2015, the proportion of fields with the higher grade of IFI was increased from 9.79% in 2000 to 27.87% in 2015, and IFI meanly increased from 0.492 in 2000 to 0.556 in 2015, increased by 13.00%. However, it should be pointed out that the soil fertility of tobacco-planting fields in Chenzhou is still in the middle level of IFI (0.4–0.6), the proportion of tobacco-planting fields with the middle grade of IFI was the highest (77.35% in 2000 and 70.52% in 2015), and there was no tobacco-plating field with the highest grade of IFI (≥0.8); therefore, the soil fertility of tobacco-planting fields in Chenzhou still needs to be promoted.

The annual fertilization for tobacco fields in Chenzhou is generally as follows from 2000 to 2015: during tobacco growing season, about 178.5 (N), 139.65 (P_2_O_5_) and 420 (K_2_O) kg/ha are applied in the forms of compound fertilizers, and during rice growing season, about 0–375 kg/ha of the compound fertilizer (N:P_2_O_5_:K_2_O = 15:15:15) and 0–75 kg/ha of urea (*N* = 46.2%) are applied according to the growing status of rice. Meanwhile, this fertilization pattern is almost the same and seldom changed from 2000 to 2015. From the improvement of tobacco planting soil, it can be seen that in 2015 there were 62.37 and 10.90% of the samples were higher and lower in pH. These tobacco-planting fields should be paid attention to the modification of soil alkalinity or acidity; 90.33, 83.13 and 64.55% of the samples were higher in OM, TN and AN, respectively, and these fields need to reduce the use of organic and nitrogen fertilizers. In Ca^2+^ and Mg^2+^, 85.12 and 44.45% of the samples were higher, respectively; these fields should be controlled by the application of alkaline substances or calcium and magnesium fertilizers. 27.61, 32.04, 12.99, 66.54 and 76.30% of the samples were lower in AK, Mg^2+^, Mn, Mo and Cl^−^, respectively; these fields should be applied with the corresponding fertilizers. Usually, tobacco is a chlorine-free crop, but chlorine is also one of the essential nutrients for tobacco growth [5], and some studies conducted in south China have shown that proper application of chlorine fertilizer to Cl-deficient soils could increase the elasticity, oiliness and yield of tobacco leaves while not reducing the quality of tobacco leaves [47,54]. Nevertheless, for the fields with higher contents of other various nutrients, such as S, Fe, Cu and Mn, there is no need to apply the corresponding fertilizers.

It should be pointed out that there is a certain relationship between soil fertility or IFI with the growth, yield and quality of tobacco [7,55,56,57,58]; it is not evaluated in this study but would be conducted in our further research.

## 5 Conclusion

This study compared the values of topsoil (0–20 cm) fertility indicators of tobacco planting area in Chenzhou in 1980s, 2000 and 2015 and found that soil pH value was decreased, TN, OM, AP, AK, Ca, Mg and Cu contents were increased, TP, S and Cl contents were decreased, and TK, B, Fe, Mn and Zn were decreased first and then an increased. In 2015, most of the samples (44.45–100%) were higher in pH, OM, TN, AN, AK, Ca^2+^, Mg^2+^, S, Fe, Mn, Cu and Zn, most of the samples (62.18%) were suitable in B, while most of the samples (59.24–79.81%) were lower in TK, Mo and Cl^−^. Most of the samples were in the middle grade of IFI (77.35% in 2000 and 70.52% in 2015), and the mean IFI was increased by 13.00% from 0.492 in 2000 to 0.556 in 2015, but both still belonged to the middle grade of IFI. Thus, soil fertility still needs to be promoted, and more attentions should be paid to modify of soil acidity and alkalinity, reduce the application of organic, nitrogen, calcium fertilizers, and increase the application of fertilizers of potassium, magnesium, molybdate and chloride according to the real situation of tobacco-planting fields.
